# Pancreatic Hydatid Cyst Causing Acute Pancreatitis: A Case Report and Literature Review

**DOI:** 10.1155/2018/9821403

**Published:** 2018-03-05

**Authors:** Bayan Alsaid, Maryam Alhimyar, Fadi Rayya

**Affiliations:** ^1^Department of General Surgery, Al-Assad University Hospital, Faculty of Medicine, Damascus, Syria; ^2^Laboratory of Anatomy, Faculty of Medicine, University of Damascus, Damascus, Syria; ^3^Faculty of Medicine, Damascus University, Damascus, Syria

## Abstract

Hydatidosis is a public health problem in endemic countries. Hydatid cysts are located usually in the liver and the lungs. Primary pancreatic hydatid cyst is in unusual location and rarely causes acute pancreatitis. In this paper, we report a case of a 34-year-old man who admitted with recurrent acute pancreatitis. Following the preoperation investigations, the primary impression was a pancreatic pseudocyst. During surgery, a primary hydatid cyst was detected in the pancreas measuring 35 × 20 × 15 cm. The treatment consisted of evacuation and external draining of the cyst. In addition, we summarized 14 cases of primary hydatid cyst of the pancreas associated with acute pancreatitis reported in the literature.

## 1. Introduction

Echinococcosis, hydatid disease, and hydatidosis are alternative names of a zoonotic parasitic disease caused by the tapeworm *Echinococcus*. It is endemic in Mediterranean countries, the Middle East, South America, and the Indian subcontinent [1–3]. Four types of *Echinococcus* lead to infection in humans; *Echinococcus granulosus* is the most common parasite causing cystic echinococcosis with larval stage that represents more than 95% of cases [4]. It can infest various organs, and the invasion to the liver and lungs accounts for 90% [5]. Other involved sites are the muscles, bones, kidneys, brain, spleen, and pancreas. Pancreatic localization is a rare situation of hydatidosis representing 0.2% of cases [6].

Acute pancreatitis is commonly associated with alcohol intake or disorders of the pancreaticobiliary tracts [7]. Acute pancreatitis has rarely been reported due to a hydatid cyst of the pancreas [8]. Only 14 cases were reported in literature.

We report a case of hydatid cyst diagnosed after acute pancreatitis episode, and we reviewed and summed the data from other reported cases of hydatid cyst manifested as acute pancreatitis in the English and French literature.

## 2. Case Presentation

A 34-year-old man was admitted to the Surgery Department in our centre in July 2016 with diffuse abdominal pain, dyspnea, general fatigue, and weakness. In his medical history, he had been admitted to another centre two months ago for an episode of acute pancreatitis.

**Figure 1:**
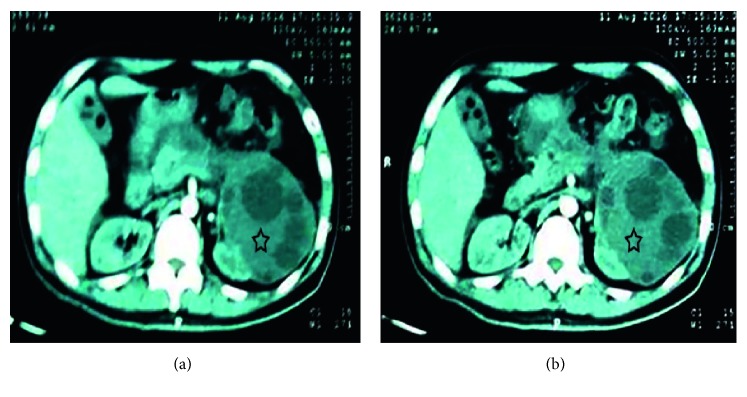
Computed tomography revealing a cystic lesion extending between the body of the pancreas and left kidney (black stars).

Abdominal ultrasonography revealed a heterogeneous area of 5 cm in size in the body of the pancreas, peripancreatic fluid, gallstones with thickness in the gallbladder wall, and multiple cysts in the left kidney. Abdominal computed tomography (CT) showed heterogeneous collection of fluid with a thick wall of 12 × 4 cm in size along the body of the pancreas and left colic angle (often an abscess or a pseudocyst) with infiltration of adipose tissue around it and mild thickness at the wall of the colon. A simple renal cyst was also reported in the left kidney (Figure 1).

Laboratory investigations were within normal levels except an elevation in C-reactive protein value (18.1 mg/dl) and amylase (765 U/L). The hepatic tests were within the normal range (total bilirubin (TB) was 0.64 mg/dl, alanine aminotransferase (ALT) was 20 IU/L, and aspartate transaminase (AST) was 19 IU/L). The patient was treated as an episode of acute pancreatitis.

Other investigations were performed; upper gastrointestinal endoscopy (UGI endoscopy) demonstrated an esophagitis (grade A) at the lower esophagus, incompetence of the lower esophageal sphincter (LES), diffuse congestion of the mucous membrane of the stomach, and aphthous ulcer at the fundus of the stomach. Lower GI endoscopy was normal until the terminal ileum.

A month later, the CT scan for thorax and abdomen data were similar to the previous finding, and the pancreatic cyst measuring 13.5 × 7 cm stretched down through the peritoneal cavity in front of the mesenteric vessels.

The laboratory values were normal, and a primary diagnosis of pancreatic pseudocyst was probable, and the decision of surgical intervention was decided.

**Figure 2:**
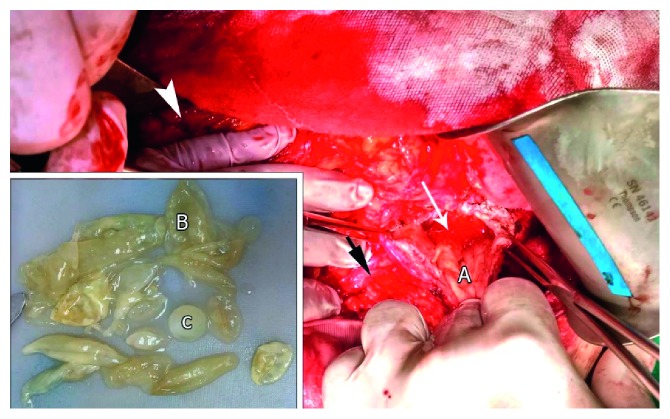
Intraoperative aspect. Pancreas (black arrow), opened cavity in the pancreas (white arrow), and stomach (arrowhead). A: cyst wall; B: endocystic membrane; C: daughter cyst.

Intraoperatively, an extreme oedema in the pylorus, the transverse mesocolon, the head and body of the pancreas, and the hepatoduodenal ligament was found. A cholecystitis required cholecystectomy. After entering the lesser sac, a large mass of 35 × 20 × 15 cm in size was found, located in the space between the tail of the pancreas, spleen, left colic angle, left kidney, stomach, and diaphragm. The mass was hard to dissect from the neighboring structures. In puncture, a clear pure liquid was aspirated proposing the existence of a hydatid cyst. Cyst fenestration was performed, and multiple daughter cysts were evacuated; the endocyst membrane was removed (Figure 2). A Foley catheter was placed in the residual cavity. The simple renal cyst needs no intervention according to the urologist. The final diagnosis was pancreatic hydatid cyst.

The patient had another episode of acute edematous pancreatitis after a month of surgery, and the amylase level was over 1000 U/L. The development of local retroperitoneal abscess required puncture and drainage by CT; the patient also developed a deep venous thrombosis and was treated by anticoagulants.

During 18 months of follow-up, the patient was well with no episodes of recurrence or other complications.

## 3. Literature Review

Besides our case, 14 cases of a pancreatic hydatid cyst with acute pancreatitis were reported. Three of them are available with abstract and one is not accessible. The ratio of women to men was 3/10. The mean age of the patients was 30.2 years. The location was solitary in the pancreas in 10 patients. The cyst was found in the body (7.1%), tail (28.5%), body and tail (21.4%), or head (28.5%) (Table 1).

On clinical examination, no specific complaints or signs were found to distinguish hydatid cyst from other etiology of acute pancreatitis so that the final diagnosis was made preoperatively in 4 cases by ultrasonography or computed tomography. In our case, the final diagnosis was made intraoperatively.

Laboratory investigations varied between cases, and they were not available in all cases. The average of white blood cells was 17,943/mm^3^, and the amylase median was 1718 U/L.

Surgical procedures that have been carried out varied according to every situation; for example, left pancreatectomy with splenectomy was performed in 5 cases. Only 2 patients were treated with albendazole before surgery.

After surgery, there were some complications such as external pancreatic fistula (*n*=1), complex pancreatic fistula (an external and enterocutaneous fistula between the transverse colon and the skin surface), and portal vein thrombosis (*n*=1). Fistulae were treated with parenteral nutrition, antibiotic therapy (ampicillin), and adequate local treatment.

The mean follow-up was 25 months without recurrence or other complications (missing value = 6) (Table 2).

## 4. Discussion

Hydatid cysts can localize virtually in any organ and structure of the body. The highest rate of cysts location exists in the liver and the lungs, which assess to 70% and 20% of patients, respectively. Other organs present in a small proportion of patients [21, 22]. It is rarely detected in the pancreas.

According to a previous review in 2012 [17], the cyst is single in the pancreas in 90% of the cases. Moreover, 50% of the cysts can be located in the head, 24–34% in the body, and 16–19% in the tail.

The clinical manifestations of cystic echinococcosis (CE) depend on the size of the cyst, site, and potential complications such as rupture, compression on adjacent tissue or organs, infections, and intestinal or biliary fistula [23].

Hepatic hydatid cysts rupture into the biliary tree in 5–17% of the cases [24]. This complication is rarely associated with acute pancreatitis [25, 26]. Hepatic hydatid cysts can also compress the biliary tract [27]. In accordance with that, we can say that the pancreatic hydatid cysts may compress Wirsung's duct or rupture into it, which produces acute pancreatitis.

Diagnosis of cystic hydatidosis is based on imaging. Ultrasonography allows classification of the cysts, as the WHO classification tabulates cysts in 3 groups: the first one is active and includes CE1, which appears as unilocular anechoic cystic lesion with double-line sign, and CE2, which is a multiseptated “rosette-like” “honeycomb” cyst; the second one is transitional and contains CE3a cyst with detached membranes (water-lily sign) and CE3b cyst with daughter cysts in the solid matrix; and finally, the third group is inactive and has CE4 and CE5 cysts with heterogeneous hypoechoic/hyperechoic contents and no daughter cysts and solid plus calcified wall [28].

CT scan demonstrates the features of hydatid cysts better than US. CT scan can identify the number, size, shape, margins, anatomic location, and calcification. Furthermore, the abdominal CT scan may be used to evaluate complications like rupture into the main pancreatic duct. It also assesses the lesions during therapy [16]. Magnetic resonance imaging (MRI) has a role in detecting the characters of the cysts and complications better than CT; however, MRI is usually not desired because of its cost [21].

The management of the cystic echinococcosis (CE) includes surgery, percutaneous treatment, antiparasitic drug therapy, and observation [29]. Surgery is the classic approach to treat CE, and it is appropriate for complicated cysts and in cases for which percutaneous treatment is not available [29]. Evacuation of the cyst and defacement of the residual cavity are the aims of the surgical therapy [30]. PAIR (puncture, aspiration, injection of a scolicidal agent, and reaspiration) is one technique of percutaneous treatment used as a therapeutic and diagnostic option that counts as an effective replacement to surgery with lower rates of recurrence and mortality [28]. Medical therapy is useful when it is combined with/to surgery and percutaneous therapy. Drug treatment before and after the procedure reduces the risk of recurrence. The first medication to be used is mebendazole and then there were albendazole and praziquantel. Albendazole is the most effective [11].

## 5. Conclusion

Despite pancreatic hydatid cyst being rare, it may be treated as a reason to acute pancreatitis. Hydatid cysts should be counted as a significant differential diagnosis for cystic lesions of the pancreas and other organs, especially in endemic regions. Clinicians must know about complications of hydatid cyst in its uncommon locations.

## Figures and Tables

**Table 1:** Summarized results of the recent review of hydatid acute pancreatitis cases.

Characteristic	Case number (%)
Age (years)	
Median (30.2)	14 (100)
Range (18–51)	
Sex	
Female	3 (21.4)
Male	10 (71.4)
Location	
Solitarily in the pancreas	11 (78.5)
Liver	1 (7.1)
Kidney	1 (7.1)
Liver and left kidney	1 (7.1)
Location in the pancreas	
Head	4 (28.5)
Body	1 (7.1)
Tail	4 (28.5)
Body + tail	3 (21.4)
Size (cm)	
Median (7.5)	7 (50)
Range (3.5–17)	
Lab investigations	
Total bilirubin (mg/l)	
Median (3.04)	3 (21.4)
Range (0.4–5.9)	
Direct bilirubin (mg/l)	
Median (1.34)	2 (14.2)
Range (0.3–3)	
Positive hydatid serology (ELISA)	4 (28.5)
White blood cells (WBCs)/mm^3^	
Median (17,943)	7 (50)
Range (11,800–24,500)	
Amylase (U/L)	
Median (1718)	8 (57.1)
Range (400–4985)	
Final diagnosis	
Preoperative	4 (28.5)
Intraoperative	4 (28.5)

**Table 2:** Literature review of hydatid acute pancreatitis cases.

Case number	Source	Year	Type of the pancreatitis	Pathogenesis	Surgical treatment	Follow-up (months)
1	Augustin et al. [9]	1984	—	Opening	Distal pancreatectomy + splenectomy	—
2	Papadimitriou [10]	1987	Edematous	Opening	Cyst fenestration	12
3	Lo Monte et al. [11]	1998	**—**	Compression	Cyst fenestration	—
4	Ozmen et al. [12]	2005	Necrotizing	Compression	Cyst fenestration + splenectomy	4
5	Pouget et al. [13]	2009	—	—	Distal pancreatectomy + splenectomy	—
6	Chammakhi-Jemli et al. [14]	2010	Necrotizing	—	Distal pancreatectomy + splenectomy	—
7	Karakas et al. [3]	2010	Edematous	Opening	Distal pancreatectomy	4
8	Diop et al. [15]	2010	Edematous	Opening	Distal pancreatectomy	48
9	Suryawanshi et al. [16]	2011	Edematous	Compression	Laparoscopic evacuation	3
10	Makni et al. [17]	2012	Edematous	Opening	Distal pancreatectomy + splenectomy	8
11	Birlutiu and Birlutiu [18]	2015	Necrotizing	—	Operculectomy	98.5
13	Mohamed et al. [19]	2015	Atrophy	—	Distal pancreatectomy + splenectomy	—
14	Mattous and Belabbes [20]	2015	—	—	Evacuation and external drainage	—
15	Our case	2017	Edematous	Compression	Evacuation and external drainage	18
